# Roles of E2F family members in the diagnosis and prognosis of head and neck squamous cell carcinoma

**DOI:** 10.1186/s12920-023-01470-6

**Published:** 2023-02-28

**Authors:** Yaoxu Li, Yinpei Huang, Bing Li, Kai Yang

**Affiliations:** 1 Department of Oral and Maxillofacial Surgery, The First Affiliated Hospital of Chongqing Medical University, No.1, Youyi Road, Yuzhong District, 400016 Chongqing, China; 2 Department of Otorhinolaryngology, The First Affiliated Hospital of Chongqing Medical University, 400016 Chongqing, China

**Keywords:** TCGA, Bioinformatics, E2Fs, HNSCC, TME

## Abstract

**Background:**

Head and neck squamous cell carcinoma (HNSCC) is the sixth most prevalent cancer worldwide. E2Fs are a group of transcription factors involved in the carcinogenesis and progression of various cancers. However, the exact roles of each member of E2F family in the development and progression of HNSCC are still unknown.

**Methods:**

RNASeq and clinical follow-up information were extracted from The Cancer Genome Atlas (TCGA). The expressions of E2Fs and their roles in HNSCC progression were explored using the R software and the cBioPortal database.

**Results:**

Our results showed that the mRNA levels of E2Fs were significantly higher in HNSCC tumors than in normal tissues. E2F1, E2F3, E2F4, E2F6, and E2F7 were identified as reliable diagnostic markers. E2Fs (except for E2F3) expressions were closely related to the clinical features (excluding metastasis) of HNSCC. High E2F6 mRNA expression was an independent risk factor for the OS of female HNSCC patients. In addition, high E2F4 expression could lead to poor prognosis in HNSCC in both males and females, high expressions of E2F5, E2F6, and E2F7 were associated with poor OS of female HNSCC patients, while high E2F2 and E2F8 expressions were positively correlated with the OS of male HNSCC patients. Interestingly, E2Fs expressions had stronger associations with immune cell infiltrations in male HNSCC patients than in female HNSCC patients.

**Conclusion:**

The expressions of E2Fs were found to be correlated with the progression of HNSCC. E2F1, E2F3, E2F4, E2F6, and E2F7 could be good diagnostic molecules for HNSCC. In addition, E2F6 was an independent risk factor for the prognosis of female HNSCC patients.

## Introduction

Head and neck squamous cell carcinomas (HNSCCs) are the most common type of head and neck cancers arising from the mucosal epithelium of the oral cavity, pharynx, and larynx [1]. It is reported that HNSCC is an important public health issue, ranking as the sixth most prevalent cancer worldwide, with an average of approximately 600,000 new cases every year [2, 3]. HNSCC is generally associated with exposure to carcinogens from tobacco and excessive alcohol consumption. Tumors derived from the oropharynx are usually associated with human papillomavirus (HPV) [4, 5]. Moreover, HNSCC (HPV^−^) differs from HNSCC (HPV^+^) in prevalence, risk factors, gene expression, molecular mechanisms, etc. [6]. Depending on the cancer stage, surgery with or without radiotherapy, chemotherapy, or other new treatments is used to treat HNSCC. However, the overall survival (OS) of HNSCC patients remains dismal, at about 50% [7, 8]. Findings from numerous epidemiological studies have partly linked the poor prognosis to delayed diagnosis [9] because most HNSCC patients are diagnosed in the late stage due to the lack of effective screening models and dependence on regular physical examination as the primary approach for early detection [10]. Although a proportion of patients with oral pre-malignant lesions (OPLs) may present with some characteristic symptoms, most patients diagnosed with advanced-stage HNSCC have no obvious pre-malignancy signs and symptoms. Therefore, significant attention is required for the early detection of HNSCC. Traditionally, clinical factors, including tumor staging, grading, and pathological feature, are helpful in predicting the prognosis of cancer patients [11]. Recent evidence suggests single molecular factors, such as the abundance of mRNA and proteins, as prognostic markers. Furthermore, molecular detection is more sensitive and faster than clinical diagnosis and has shown promising efficacies for the early diagnosis of cancer [12]. However, there is still a lack of reliable and clinically useful predictive or prognostic biomarkers for diagnosing HNSCC.

E2Fs are a group of transcription factors encoded by a family of genes with highly homologous and conserved DNA binding and retinoblastoma (RB) protein-binding domains [13–15]. This transcription factor family comprises nine identified members reported to be involved in cell cycle regulation and DNA synthesis. Traditionally, E2F1, E2F2, and E2F3a were identified as activators, while E2F3b, E2F4, E2F5, E2F6, E2F7, and E2F8 as repressors. Later, E2F4 was also found to be a transcriptional activator. RB proteins can inhibit E2Fs in normal cells, with the RB-E2F pathway reported to be regulated by cyclin/cyclin-dependent kinase (CDK) complexes, cyclin kinase inhibitors (CKIs), and the E7 protein of the HPV virus. Specifically, cyclin/CDK complexes can inactivate RB through phosphorylation, which can be inhibited by CKIs, and the E7 protein can specifically bind to and degrade RB. Subsequently, both phosphorylation and degradation of RB can lead to the upregulation of E2F-responsive transcription to promote cell cycle and cell proliferation [16, 17]. The deregulated E2F activity induced by the functional inactivation of RB Transcriptional Corepressor 1 (RB1) was found to be associated with aberrant cell proliferation and other cancer-related biological processes in various human cancers [18, 19]. Moreover, an increasing number of studies have demonstrated that the abnormal expression of E2Fs is associated with various cancers, including those of the breast, ovaries, stomach, brain, etc. [20–22]. Notably, E2Fs were discovered to have dual roles in tumor promotion and suppression [23]. It was found that E2Fs might influence oral squamous cell cancer (OSCC) and that high expression of E2F gene sets was associated with a worse prognosis [24]. Another study revealed that the E2F-regulated genes were upregulated in most HPV-positive HNSCC and a set of HPV-negative HNSCC [25]. In addition, E2F2 polymorphisms could be used to predict the risk of recurrence of oropharyngeal squamous cell carcinoma [26]. Nevertheless, few studies have investigated the exact role of each E2F member in the development and progression of HNSCC.

The developments of microarray and sequencing technologies for analyzing multiple databases have revolutionized biological and biomedical studies and made RNA and DNA research more efficient [27]. To the best of our knowledge, there is inadequate literature on bioinformatics analysis exploring the role of E2Fs in HNSCC. This present study aimed to assess the expression patterns, investigate the distinct prognostic significance, and determine the potential functions of E2Fs in HNSCC. Specifically, we used hundreds of gene expressions or variations from several large public databases to analyze the expressions and mutations of E2Fs and the associated clinical parameters, prognostic prediction functions, immune infiltrations, and molecular pathways in HNSCC patients.

## Materials and methods

### mRNA expression of E2Fs and clinical characteristics in TCGA

The Cancer Genome Atlas (TCGA) is publicly available and open-ended, based on which we retrieved RNASeqV2 data from the TCGA-HNSC (head and neck squamous cell carcinoma) project (https://tcga.xenahubs.net) as previously described [28]. Approval from our local ethics committee was not required. The data of 502 head and neck cancer tissues and 44 adjacent tissues were available in the HNSCC patient cohort. The expression levels of E2Fs mRNA, general information, and clinicopathological details were extracted. Then, the mRNA expressions of E2Fs in HNSCC patients were analyzed and compared with that of normal or adjacent non-tumor tissues. Additionally, the correlation between mRNA expression of E2Fs and clinical features, including the tumor, node, and metastasis (TNM) stages, gender, age, histologic grade, and smoking, were analyzed. Further, gender-disaggregated COX regression, Kaplan–Meier, Receiver Operating Characteristic (ROC) curve, and immune infiltration analyses were conducted using the R statistical software.

### Cell culture and quantitative real-time PCR (qRT-PCR)

The human oral mucosal HOK cells were purchased from Shanghai Bayley Biotechnology Co., Ltd. (Shanghai, China). The oral squamous cell carcinoma cell lines SCC15 and TSCCA were purchased from Shanghai Zhongqiao Xinzhou Technology Co., Ltd. (Shanghai, China). HOK, SCC15, and TSCCA cells were grown in Dulbecco’s modified Eagle’s medium (DMEM, Gibico, USA) supplemented with 10% fetal bovine serum (FBS, PAN- Biotech, Germany) and 1% penicillin-streptomycin at 37 °C with 5% CO2. Total RNA was isolated using an RNA Isolation Total RNA Extraction Reagent (R401-01, Vazyme, China) according to the manufacturer’s instructions. RNA was reverse transcribed into cDNA using an RT Master Mix for qPCR II (gDNA digester plus) kit (HY-K0511A, MedChemExpress, USA). RT-PCR was conducted on the CFX Connect Real-Time System (1,855,201, BIO-RAD, USA) using an SYBR Green qPCR Master Mix (No ROX) (HY-K0523, MedChemExpress, USA). The experiments were conducted in more than three independent replicates. The primers for PCR analysis are listed in Table 1.

**Table 1:** Primer sequences of E2Fs and GAPDH for qRT-PCR

Genes	Forward primers	Reverse primers
E2F1	CCGTGGACTCTTCGGAGAACT	GGTTCTTGCTCCAGGCTGAGT
E2F2	TCGGTATGACACTTCGCTGGG	AACATTCCCCTGCCTACCCAC
E2F3	CCGCTTCCAAAGACTTGGCT	CATCGAAGAGATCGCTGATGCC
E2F4	GGACCCAACCCTTCTACCTCCT	CCGAGCTCATGCACTCTCGT
E2F5	GGGCTGCTCACTACCAAGTTC	CCAGCACCTACACCTTTCCAC
E2F6	AGCATTCAGGCCTTCCATGAAC	GCACTGTGATAGAGTCTTCTCTGG
E2F7	ACCCGACTGTCCCTCTTCATC	CAGAGCCAAGCTGGTCAGAAC
E2F8	CCTGAGATCCGCAACAGAGAT	AGATGTCATTATTCACAGCAGGG
GAPDH	TCAAGAAGGTGGTGAAGCAGG	AGCGTCAAAGGTGGAGGAGTG

### Genetic alteration analysis in cBioPortal

cBioPortal was used as previously described (https://www.cbioportal.org/) [29]. The HNSCC (TCGA, Firehose Legacy) dataset, including 530 samples from 528 cases with pathology reports, was selected to analyze the genetic alteration of E2F6 in HNSCC. Remarkably, the alteration of genes (mutations and putative copy-number alterations from GISTIC), mRNA expression z-scores (RNA Seq V2 RSEM), and protein expression z-scores (RPPA) were included and the z-score threshold of both mRNA and protein expression was settled at ± 2.0. Eventually, the results of the “Oncoprint”, “Cancer Types Summary”, and “Co-expression” modules were calculated and downloaded.

### Correlated genes and gene enrichment analysis

As described above, the top 200 expression-correlated genes of E2F6 in HNSCC were obtained by utilizing the “co-expression” module of the cBioportal database, with the same parameters described above. Moreover, the correlations between E2F6 expressions with the top 5 expression-correlated genes were further explored via Spearman correlation analysis. Subsequently, the top 200 expression-correlated genes were input to operate gene enrichment analysis. Specifically, the Kyoto encyclopedia of genes and genomes (KEGG) pathway analysis and Gene Ontology (GO) enrichment analysis of correlated genes of E2F6 was performed using the R statistical software.

### Statistical analysis

The R statistical software (v3.6.3, R Core Team) was used for statistical analyses. The significance threshold of the *P*-value was 0.05 (ns for *P* ≥ 0.05, * for *P* < 0.05, ** for *P* < 0.01, and *** for *P* < 0.001 respectively). ROC, COX regression, Kaplan–Meier, and immune infiltration analyses were performed using the “pROC”, “survival”, “survminer”, and “GSVA” R packages. Enrichment analyses were performed using the “org.Hs.eg.db” and “clusterProfiler” R packages. The visualizations were conducted by the “ggplot2” R package.

## Results

### Clinical characteristics of HNSCC patients

The clinical characteristics and gene expression data of 502 HNSCC primary tumors and 44 adjacent normal samples were downloaded from the TCGA database. The patients’ clinicopathological details, including age, gender, TNM stages, clinical stage, histologic grade, smoking, and HPV status are summarized in Table 2.

**Table 2:** Characteristics of HNSCC patients based on TCGA

Characteristics	Variable	Number of cases	Percentages (%)
Age	<=60	245	48.9
	> 60	256	51.1
Gender	Female	134	26.7
	Male	368	73.3
T stage	T1	33	6.8
	T2	144	29.6
	T3	131	16.9
	T4	179	36.8
N stage	N0	239	49.8
	N1	80	16.7
	N2	154	32.1
	N3	7	1.5
M stage	M0	472	99
	M1	5	1
Clinical-stage	Stage I	19	3.9
	Stage II	95	19.5
	Stage III	102	20.9
	Stage IV	272	55.7
Histologic grade	G1	62	12.8
	G2	300	62.1
	G3	119	24.6
	G4	2	0.4
Smoker	No	111	22.6
	Yes	381	77.4
HPV status	Negative	80	66.1
	Positive	41	33.9

### Differential Expressions of E2Fs in HNSCC

**Fig. 1:**
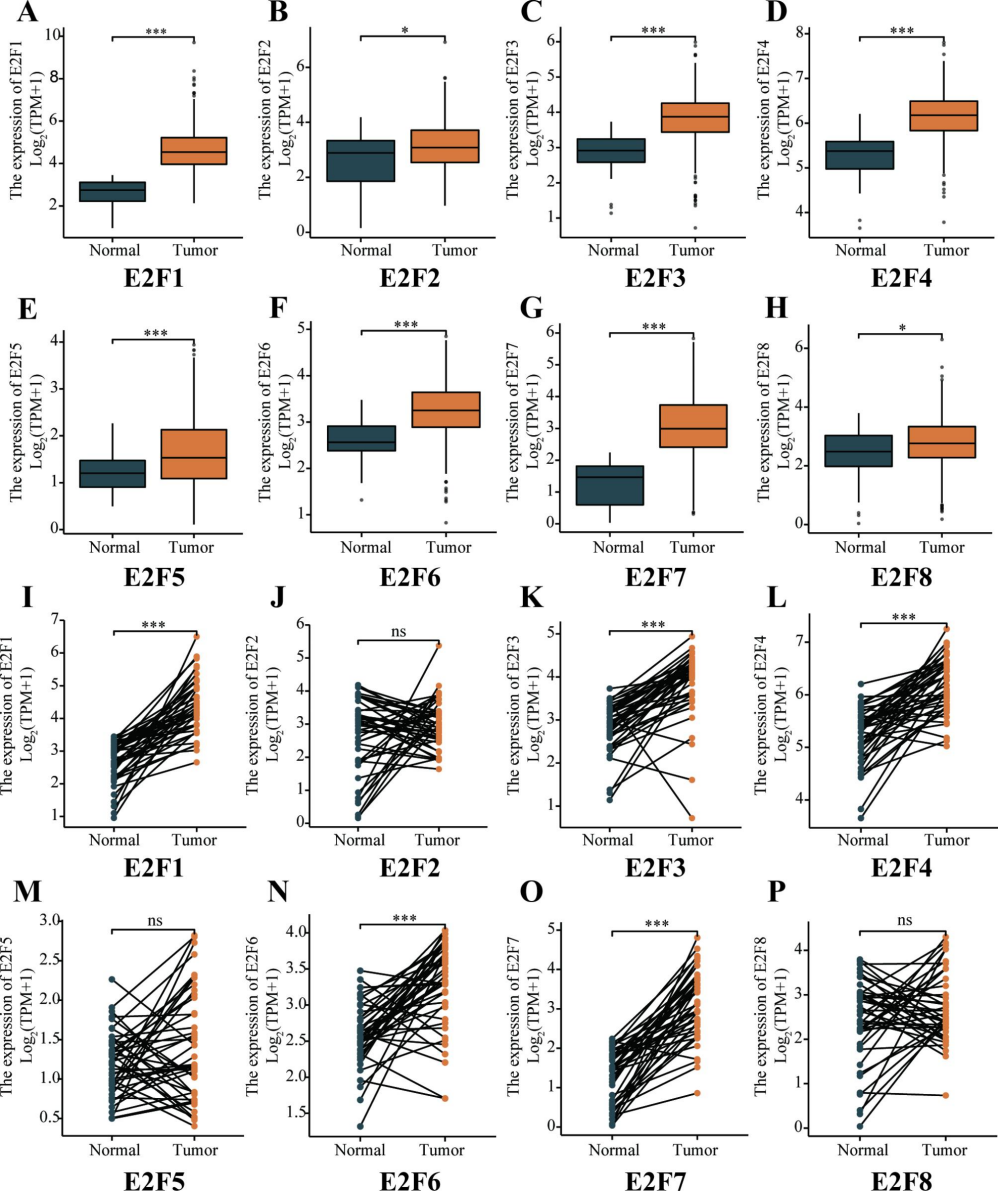
**E2Fs mRNA expression in HNSCC patients and normal samples.** The unpaired comparison for (A) E2F1, (B) E2F2, (C) E2F3, (D) E2F4, (E) E2F5, (F) E2F6, (G) E2F7, and (H) E2F8. The pairwise comparison for (I) E2F1, (J) E2F2, (K) E2F3, (L) E2F4, (M) E2F5, (N) E2F6, (O) E2F7, and (P) E2F8.

**Fig. 2:**
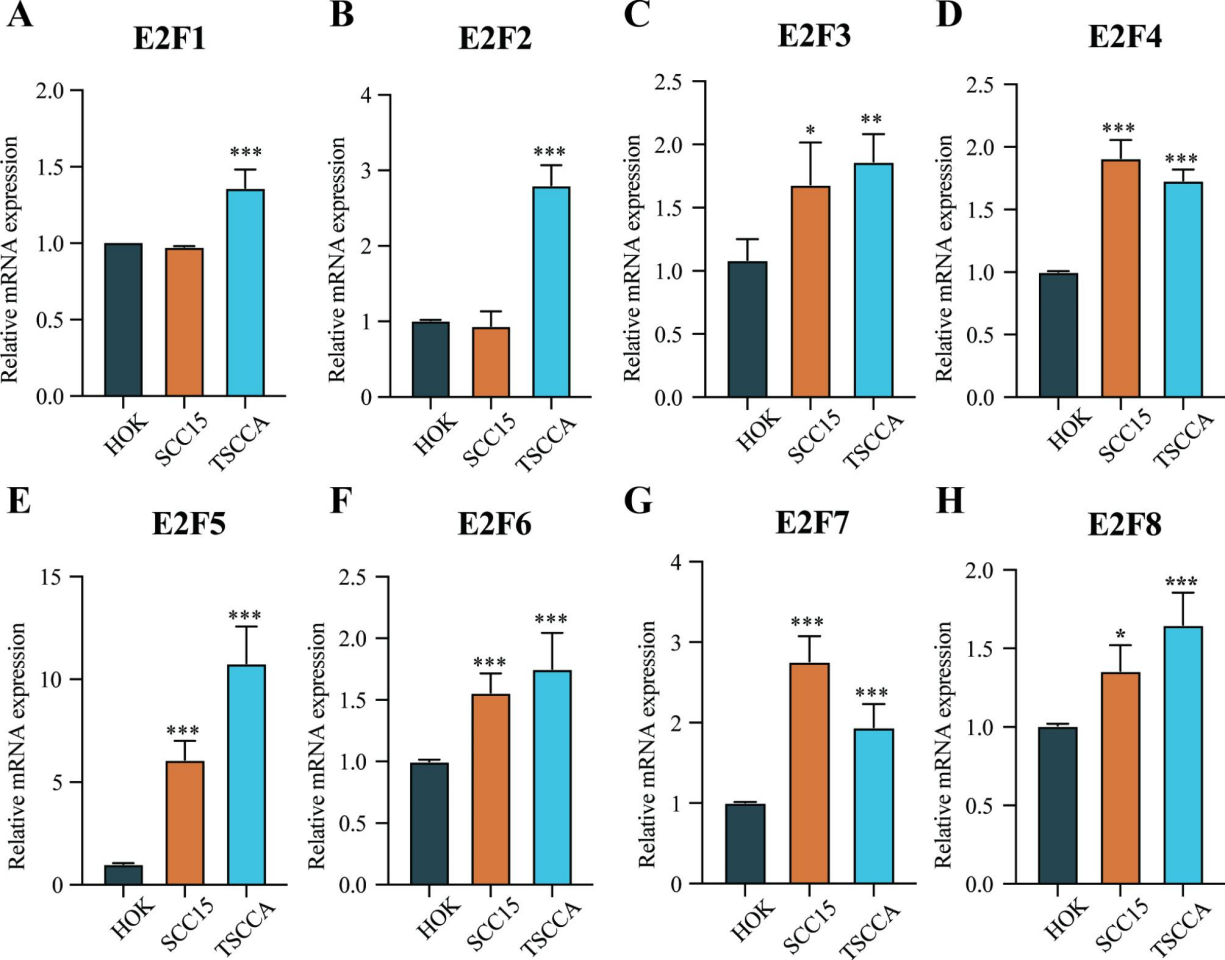
**E2Fs mRNA expression in human oral mucosal HOK cells and oral squamous cell carcinoma cell lines SCC15 and TSCCA.** (A) E2F1, (B) E2F2, (C) E2F3, (D) E2F4, (E) E2F5, (F) E2F6, (G) E2F7, (H) E2F8. Mean ± s.e.m.; *P < 0.05, **P < 0.01, ***P < 0.001 compared with HOK; Student’s t-tests. These data are representative of three independent experiments

The transcription levels of E2Fs were analyzed using the TCGA database. In the unpaired comparison, we found that the mRNA expressions of all E2Fs in HNSCC patients were significantly higher than those of normal tissues (*P* < 0.05) (Fig. 1A-H) and the differences were more significant in E2F1, E2F3, E2F4, E2F5, E2F6, and E2F7 (*P* < 0.001). Moreover, in the paired comparison, the expressions of E2F1, E2F3, E2F4, E2F6, and E2F7 in HNSCC patients were significantly higher than those of adjacent normal samples (*P* < 0.001) (Fig. 1I-P). However, there was no significant difference in E2F2, E2F5, and E2F8 expressions between the tumor group and the paired normal tissue group (*P* > 0.05). Figure 1 demonstrates that the expressions of E2Fs were significantly higher in HNSCC tumors than in normal tissues. Additionally, the results from qRT-PCR indicated that all E2Fs were overexpressed in HNSCC cell lines (Fig. 2 C-H), except for there was no difference in E2F1 and E2F2 expression between SCC15 and HOK cells (*P* > 0.05) (Fig. 2A-B).

### Correlations between E2Fs expression and clinical characteristics of HNSCC patients

**Fig. 3:**
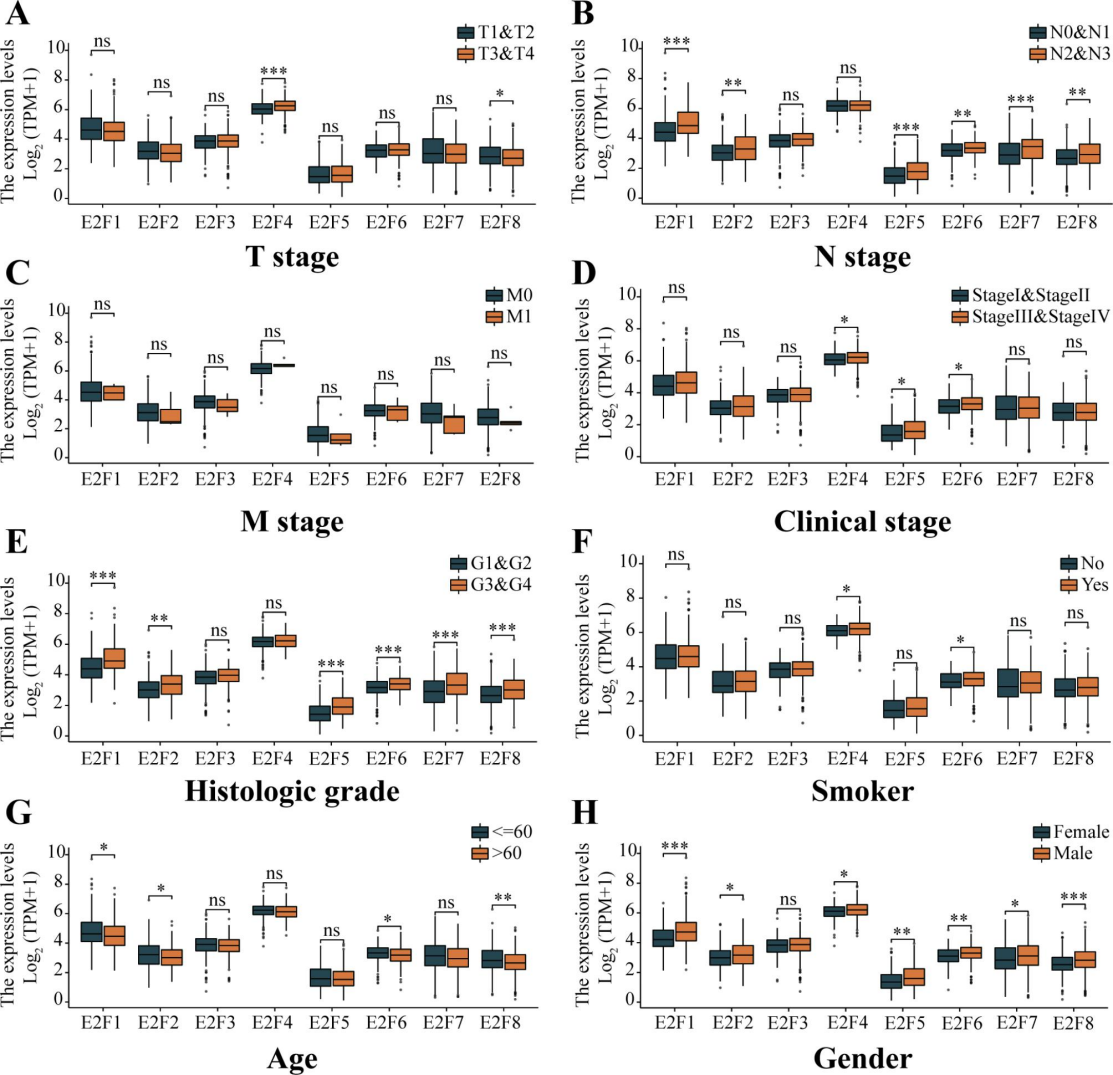
**Association of E2Fs expressions with the clinicopathologic characteristics of HNSCC patients.** (A) T stage, (B) N stage, (C) M stage, (D) Clinical stage, (E) Histologic grade, (F) Smoking, (G) Age, and (H) Gender

Figure 3 shows a strong association between E2F expression and HNSCC patients’ clinical characteristics, including TNM stages, clinical stage, histologic grade, smoking, age, and gender. However, E2F3 expression was not correlated with the clinical characteristics and tumor progression of HNSCC. Briefly, the expressions of E2F1, E2F2, E2F5, E2F6, E2F7, and E2F8 in the N2 and N3 stages were significantly higher than those in the N0 and N1 stages (Fig. 3B). Similarly, excluding E2F3 and E2F4, the expressions of E2Fs in G3 and G4 grades were significantly higher than those in G1 and G2 grades (Fig. 3E). Except for E2F3, the expressions of E2Fs were higher in males than in females (Fig. 3H). However, there was no significant association between E2Fs expressions and M stage (*P* > 0.05) (Fig. 3C). These results indicated that E2Fs (except E2F3) could potentially be novel biomarkers for HNSCC diagnosis. In addition, COX regression, ROC curve, Kaplan–Meier, and infiltration analyses were conducted disaggregated by gender as almost all the expressions of E2F genes were different in HNSCC patients of different genders (Fig. 3H).

### Effect of E2Fs on the prognosis and diagnosis of HNSCC patients

**Fig. 4:**
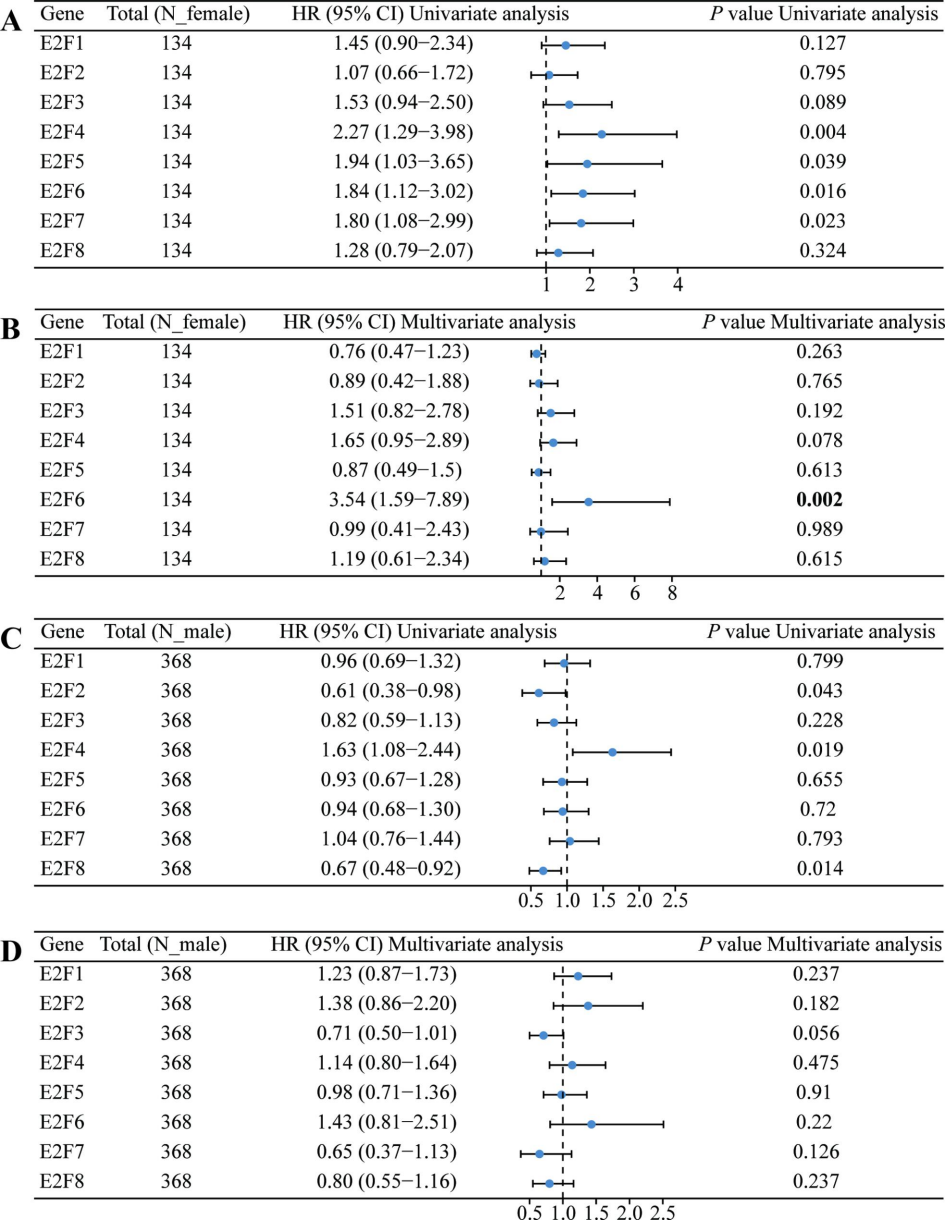
**COX regression analyses of correlations between overall survival and E2Fs expressions (High vs. Low).** Univariate analysis in (A) females and (C) males, multivariate analysis in (B) females and (D) males

COX regression analyses were conducted to explore the role of E2Fs on the prognosis of HNSCC patients. First, univariate analysis revealed that E2F4, E2F5, E2F6, and E2F7 were associated with overall survival (OS) of female HNSCC patients (all HR > 1, *P* < 0.05) (Fig. 4A). Moreover, E2F2 (HR < 1), E2F4 (HR > 1), and E2F8 (HR < 1) were significantly correlated with OS in univariate analysis of male HNSCC patients (*P* < 0.05) (Fig. 4C). Notably, only E2F6 was significantly associated with OS in multivariate analysis in females (HR > 1, *P* < 0.05) (Fig. 4B). However, no molecular was significantly correlated with OS in multivariate analysis in males (*P* > 0.05) (Fig. 4D). The results showed that high E2F6 mRNA expression was an independent risk factor for the OS of female HNSCC patients.

**Fig. 5:**
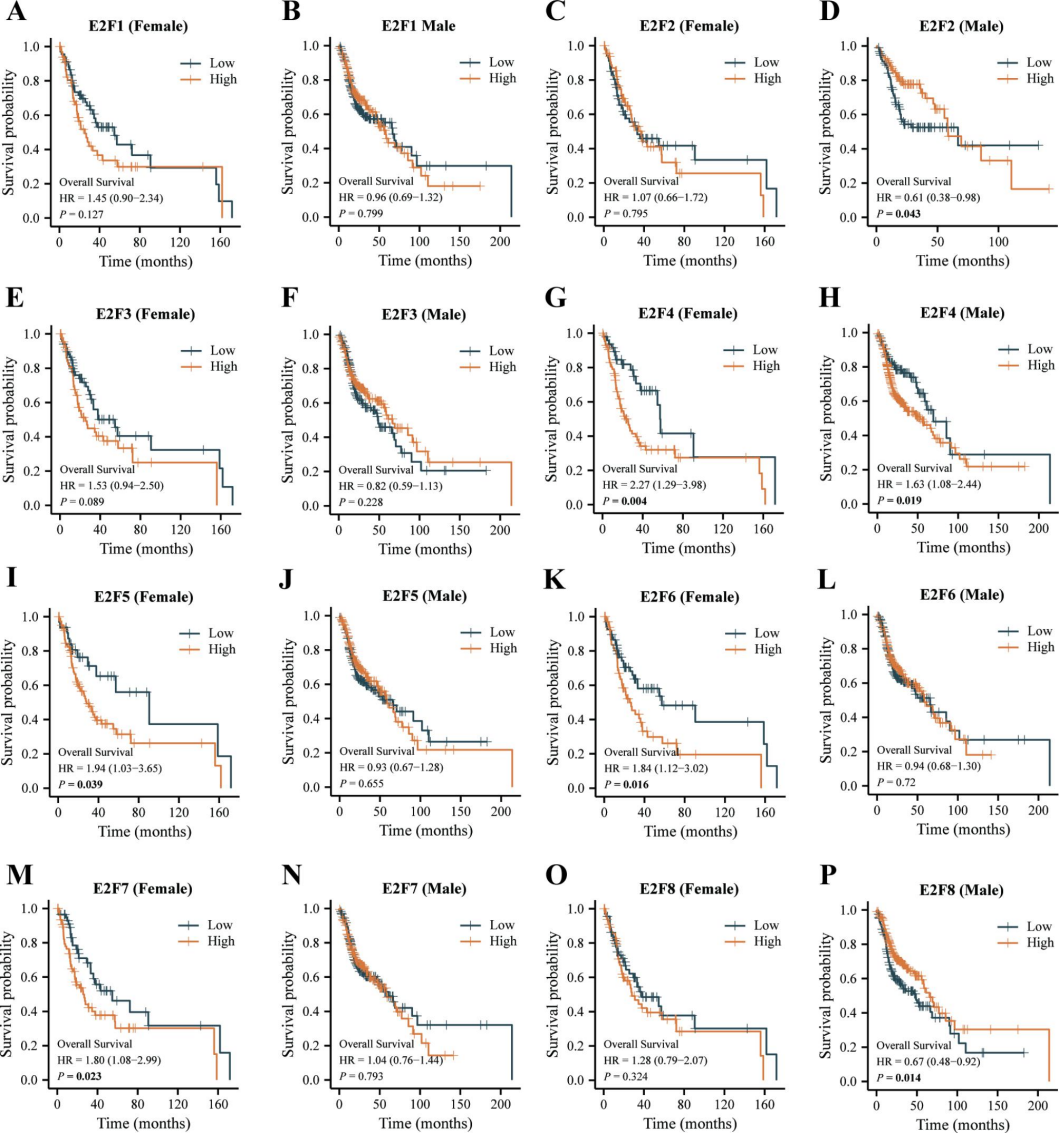
**Effects of E2Fs on the OS of HNSCC patients segregated by gender.** E2F1 of (A) female and (B) male, E2F2 of (C) female and (D) male, E2F3 of (E) female and (F) male, E2F4 of (G) female and (H) male, E2F5 of (I) female and (J) male, E2F6 of (K) female and (L) male, E2F7 of (M) female and (N) male, and E2F8 of (O) female and (P) male

Then gender-disaggregated Kaplan–Meier analyses for OS showed that high E2F4 expression could lead to poor prognosis in both male and female HNSCC patients (Fig. 5G-H). High expressions of E2F5, E2F6, and E2F7 were associated with poor the OS of female HNSCC patients (Fig. 5I, K, and M). Moreover, E2F2 and E2F8 expressions were positively correlated with the OS of male HNSCC patients (Fig. 5D and P). However, the rest results of Kaplan–Meier analyses for OS were not statistically significant (Fig. 5A-C, E-F, J, L, N, and O).

**Fig. 6:**
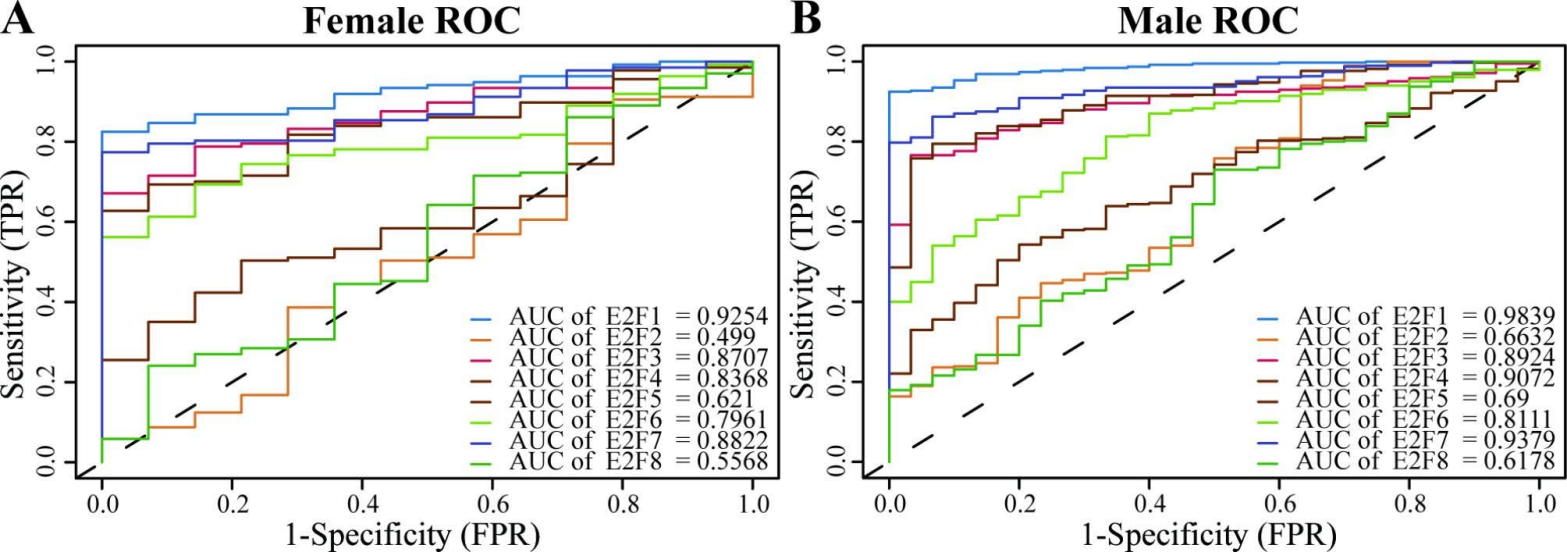
**ROC curves of E2Fs expressions in normal and tumoral HNSCC tissues in (A) females and (B) males**

Subsequently, the diagnostic significance of E2F mRNA expression in HNSCC was evaluated via ROC curve analysis in normal and tumoral tissues (Fig. 6). In female HNSCC patients, the area under the curve (AUC) of E2F1, E2F3, E2F4, E2F6, and E2F7 were above 0.75, indicating good diagnostic significance (Fig. 6A). Moreover, in male HNSCC patients, the AUC of E2F1, E2F3, E2F4, E2F6, and E2F7 were above 0.80 (Fig. 6B). The results showed that E2F1, E2F3, E2F4, E2F6, and E2F7 were good diagnostic molecules in both female and male HNSCC patients. However, the AUCs of E2F2, E2F5, and E2F8 were not robust enough for consideration.

### Correlation of E2Fs with immune infiltration in HNSCC

**Fig. 7:**
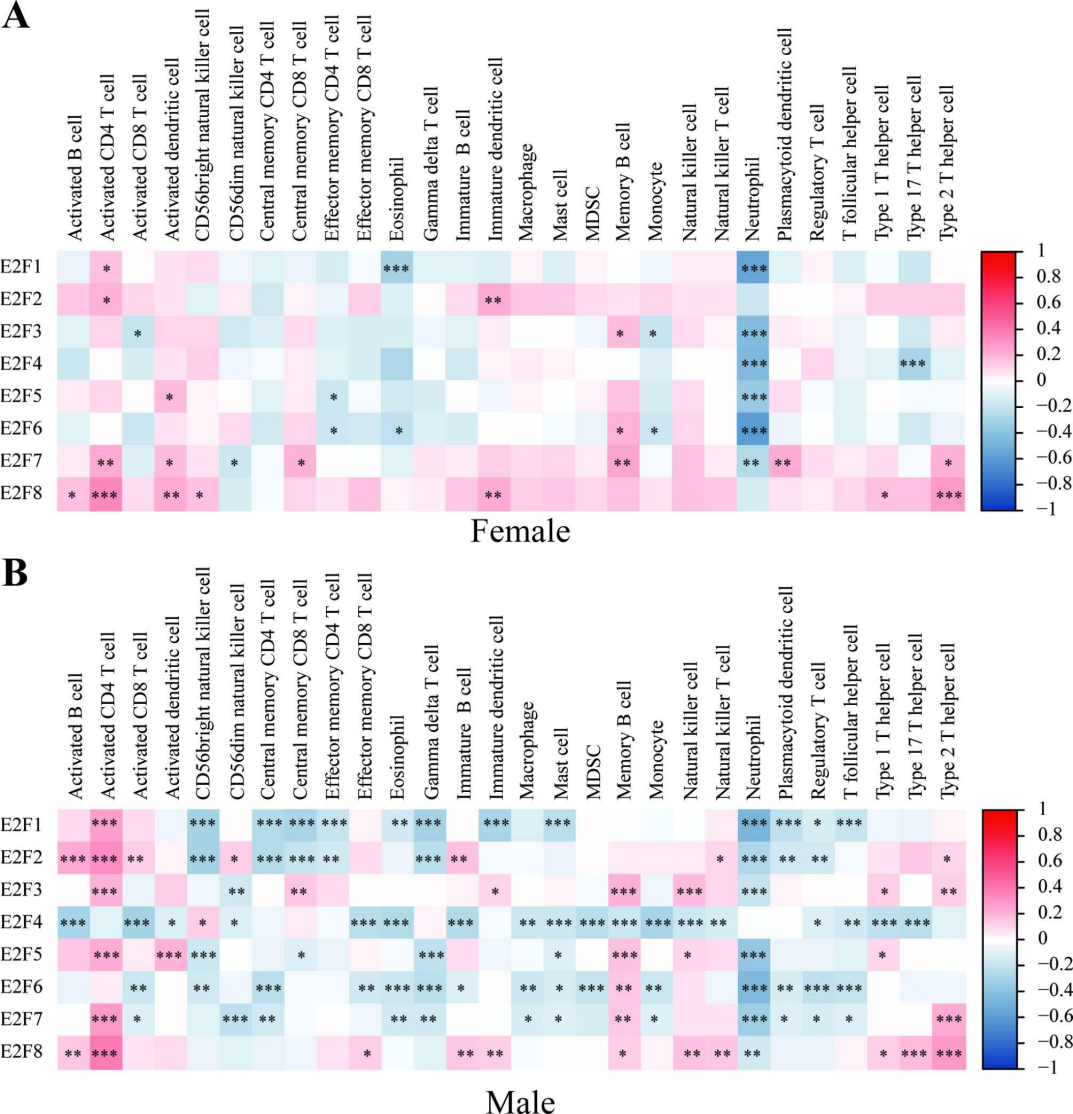
**Correlations of E2Fs expressions with the infiltration levels of immune cells in (A) female and (B) male HNSCC patients**

Tumor-infiltrating immune cells affect the survival of cancer patients [30]. Therefore, the associations of E2Fs expressions with infiltrating immune cells infiltrations in HNSCC were analyzed (Fig. 7**)**. Briefly, the correlation between immune cell infiltrations and E2Fs seemed to be stronger in males than in females. Most E2Fs expressions could be negatively associated with the infiltration level of neutrophils in both female (excluding E2F2 and E2F8) and male HNSCC (excluding E2F4) patients (*P* < 0.01). In males, E2F2 and E2F8 expression might be positively associated with infiltration levels of activated B cells, activated CD8 + T cells, effector memory CD8 + T cells, immature B cells, natural killer (NK) T cells, etc. However, a negative trend was found in most correlations between E2F4 expression and infiltration levels of all immune cells (Fig. 7B). Although these results were modest, they provided some insights into the correlation between immune infiltration and E2Fs expressions in HNSCC, especially in males.

### E2F6 genetic alterations and co-expressed genes in HNSCC

**Fig. 8:**
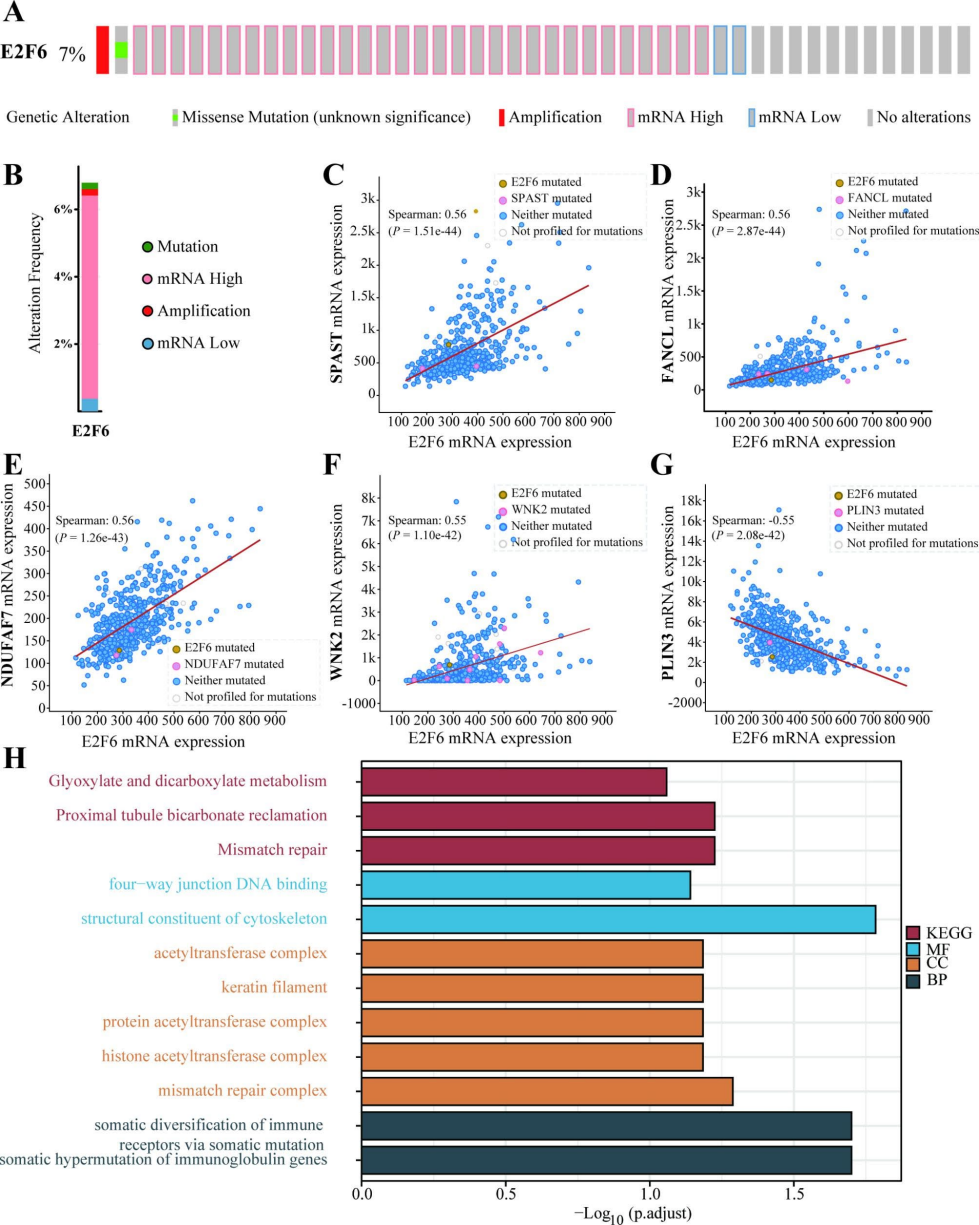
**Genetic alteration and co-expressed genes of E2F6 in HNSCC.** (A) The “OncoPrint” and (B) “Cancer Types Summary” schematics of E2F6 gene alteration. The top 5 expressed-correlated genes of E2F6: (C) *SPAST*, (D) *FANCL*, (E) *NDUFAF7*, (F) *WNK2*, and (G) *PLIN3*. **And** (H) **distributions of E2F6-associated pathways in HNSCC.**

Considering high E2F6 mRNA expression was the only independent risk factor for the OS of female HNSCC patients in the multivariate analysis, our subsequent analyses mainly focused on E2F6’s influence on HNSCC. Corresponding genetic alterations status and the top 200 expression-correlated genes of E2F6 in HNSCC were determined using the cBioPortal database. As shown in Fig. 8A and B, in the “OncoPrint” and “Cancer Types Summary” schematics, the gene alteration of E2F6 was 7% in the selected HNSCC samples. The genetic alteration of E2F6 was involved in mutation, amplification, mRNA high, and mRNA low. In addition, the primary type of E2F6 genetic alteration was mRNA high, which was consistent with the previous high expression of E2F6 in HNSCC (Fig. 1).

Next, we investigated the correlations between the mRNA expression of E2F6 and the co-expressed genes in HNSCC. As shown in Fig. 8, the expressions of the following 5 genes were most related to E2F6 expression: Spastin (*SPAST*), FA Complementation Group L (*FANCL*), NADH:Ubiquinone Oxidoreductase Complex Assembly Factor 7 (*NDUFAF7*), WNK Lysine Deficient Protein Kinase 2 (*WNK2*), and Perilipin 3 (*PLIN3*) (Fig. 8C-G). The mutation characteristics of E2F6 and genes co-expressed with E2F6 in HNSCC implied a close co-relationship between E2F6 gene expression with other genes expression, some related pathways as well.

### Enrichment analysis of correlated genes of E2F6

To further investigate the relevant molecular mechanism of E2F6 associated with the survival of HNSCC patients, the top 200 co-expressed genes associated with E2F6 in HNSCC were used to perform KEGG and GO pathway enrichment analyses.

KEGG and GO analyses on the genes co-expressed with E2F6 in HNSCC revealed 3 pathways, 2 valid molecular functions (MF), 5 cellular components (CC), and 2 biological processes (BP; Fig. 8H). Specifically, the KEGG results suggested that “Glvoxylate and dicarboxylate metabolism”, “Proximal tubule bicarbonate reclamation”, and “Mismatch repair” pathways might be associated with the effects of E2F6 on HNSCC. In MF analysis, “four-way junction DNA binding” and “structural constituent of cytoskeleton” was significantly correlated with E2F6 function. In the CC subgroup, “acetyltransferase complex”, “keratin filament”, “protein acetyltransferase complex”, “histone acetyltransferase complex”, and “mismatch repair complex” were significantly associated with E2F6 gene regulation. The involved BPs included “somatic diversification of immune receptors via somatic mutation” and “somatic hypermutation of immunoglobulin genes”. These results collectively demonstrated that E2F6 could regulate genes related to mismatch repair, acetyltransferase complex, and somatic mutation in HNSCC.

## Discussion

Our present study showed that the mRNA expressions of E2Fs were up-regulated in the TCGA-HNSCC database, which was consistent with the activation of the E2F signaling pathway in patients with OSCC recurrence after radiotherapy in a previous study [24]. The dysregulated E2F1, E2F2, E2F4, E2F5, E2F6, E2F7, and E2F8 expressions were correlated with clinical characteristics including T stage, N stage, clinical stage, gender, age, histologic grade, and smoking, indicating an extensive influence of E2Fs in the genesis and progress of HNSCC. Meanwhile, the relatively high AUCs of E2F1, E2F3, E2F4, E2F6, and E2F7 suggested good diagnostic values in both females and males. However, the expression level of E2F3 showed no substantial correlation with the clinical features involved in this study. Additionally, the M stage of HNSCC patients was not associated with the expression levels of all E2Fs. These findings indicate that E2F1, E2F3, E2F4, E2F6, and E2F7 could be reliable diagnostic molecules. Moreover, the mRNA expressions of E2Fs (excluding E2F3) were closely related to the clinical features of HNSCC, despite that E2Fs might not be effective in predicting metastasis in HNSCC. The results of gender-disaggregated survival analyses showed that high E2F4 expression could lead to poor prognosis in both male and female HNSCC patients, high expressions of E2F5, E2F6, and E2F7 were associated with poor OS of female HNSCC patients, while high E2F2 and E2F8 expressions were positively correlated with the OS of male HNSCC patients. Therefore, it seemed that E2F expression has a stronger predictive effect on the prognosis of women than men. Moreover, according to the results of analyses of COX regression analyses, E2F6 was found to be an independent risk factor for the survival of female HNSCC patients.

The tumor microenvironment (TME) in HNSCC consists of tumor cells, endothelial cells, CAFs, and immune cells. Generally, HNSCC has a high degree of immune infiltration, although the extents and compositions of immune cell infiltration could be different in different anatomic sites and etiology. Our study showed the degree of E2Fs-correlated immune infiltration in male HNSCC patients was higher than in female HNSCC patients. Importantly, it was reported that elevated levels of B cells, CD8^+^ cells, and natural killer (NK) cells generally correspond to improved survival in HNSCC [31]. Our results showed the positive regulation of E2F2 and E2F8 but the negative regulation of E2F4 on B cells, CD8^+^ cells, and NK cells in males and this could be correlated with the suppressive function of E2F4 but improved effects of E2F2 and E2F8 on the OS of male HNSCC patients. Interestingly, the infiltration level of neutrophils was negatively associated with most E2Fs expressions in both females and males. This result indicated that neutrophil infiltration might be related to the regulation of the tumor cell cycle.

Genetic alteration, co-expressed genes, and enrichment analyses were performed to explore the molecular mechanisms and determine the molecular evidence that could support the effects of E2F6 on the prognosis of HNSCC patients. Our results showed that genes related to mismatch repair, acetyltransferase complex, and somatic mutation could be regulated by E2F6 expression. As reported, mismatch repair deficiency could promote hypermutability and lead to the phenomenon called microsatellite instability. Therefore, it could affect cell proliferation and increases the probability of tumor development [32]. Meanwhile, Somatic mutations are more common than genetic mutations and most cancers are closely related to somatic mutations [33]. Hence, we speculated that up-regulated E2F6 may lead to mismatch repair deficiency and increase the frequency of somatic mutations, resulting in the progression of HNSCC. Moreover, E2F6 was proven to repress a set of tumor suppressors including BRCA1 genes via covalent histone modification [34, 35]. Combined with the CC enrichment analysis results, E2f6-related histone modification may also promote the progression of HNSCC by inhibiting the expression of tumor suppressor genes.

There were some limitations in our study that should be addressed. First, although our COX regression and survival analyses showed the potential role of E2F6 in female HNSCC patients, the effects of all genes of the E2F family in HNSCC should be more extensively studied. Second, gender-disaggregated survival analyses indicated that E2Fs might play different roles in patients of different genders. Thus, more detailed research disaggregated by gender needs to be carried out in the future. Third, infiltration analysis results suggested that E2Fs expressions had stronger associations with immune cell infiltrations in male HNSCC patients. However, the effects of E2Fs expressions on the immune microenvironment in HNSCC patients with different genders and the specific reasons remained unclear. Lastly, the effects of E2F6 on the progression of HNSCC could be partially interpreted in the genetic alterations and GO/KEGG enrichment analysis and the specific underlying regulatory mechanisms and molecular pathways involved need to be explored and verified.

In conclusion, our results suggested that the mRNA expressions of E2Fs were significantly correlated with the carcinogenesis and progression of HNSCC. More specifically, E2F1, E2F3, E2F4, E2F6, and E2F7 might be reliable diagnostic molecules, and E2Fs (excluding E2F3) were closely related to the clinical features (except for metastasis) of HNSCC. We found that E2F6 was an independent risk factor for the survival of female HNSCC patients. In addition, E2F expression has a stronger predictive effect on the prognosis of women than men in HNSCC.

Altogether, this study provided new insights into the early diagnosis and prognostic prediction of HNSCC, which could be of great significance in improving the detection rate, treatment, and prognosis estimation of HNSCC patients.

## Data Availability

The raw datasets used in this work can be downloaded at The Cancer Genome Atlas (https://portal.gdc.cancer.gov/projects/). Genetic alteration analysis was operated in the cBioPortal database (https://www.cbioportal.org/). All the databases above are open access and can be login in directly without relevant accession numbers.
